# Microscopic Colitis: A Review Article

**DOI:** 10.7759/cureus.47150

**Published:** 2023-10-16

**Authors:** Khalid I AlHussaini

**Affiliations:** 1 Department of Internal Medicine, Imam Mohammad Ibn Saud Islamic University (IMSIU), Riyadh, SAU

**Keywords:** inflammatory bowel disease, lymphocytic colitis, collagenous colitis, diarrhea, microscopic colitis

## Abstract

Microscopic colitis (MC) is a chronic inflammatory disease that affects the older population. Its clinical presentation includes a variety of gastrointestinal manifestations. The main symptom is chronic watery, nonbloody diarrhea. The disease has a female predominance. The diagnosis might be challenging since the symptoms are similar to other differential diagnoses, such as celiac disease, irritable bowel syndrome, Crohn’s disease, bacterial overgrowth, and infectious colitis. The golden diagnostic tool for diagnosis is performing colonoscopy to obtain the colonic biopsy, which demonstrates the characteristic histological evidence needed for diagnosis. The treatment starts with an accurate diagnosis and trial of any possible offending medications. Alternatively, there are many medications, such as bismuth or budesonide, which are very effective in treating this disease. The primary objective of this detailed review is to enhance knowledge and understanding of this condition among healthcare providers to guide them with detailed information regarding epidemiology, clinical presentation, diagnosis, and appropriate management. In the assessment of individuals presenting with persistent chronic diarrhea, it is essential for healthcare providers to consider MC as a probable differential diagnosis.

## Introduction and background

Microscopic colitis (MC) is considered a common cause of persistent watery diarrhea in elderly people [1]. It is a chronic inflammatory disease of the large intestine, primarily affecting individuals in the middle-aged demographic, with a higher prevalence among females [2]. The large intestine exhibits a normal mucosal morphology on colonoscopy in people with MC. The accurate diagnosis is confirmed by performing a colonoscopy with random colonic biopsies showing the characteristic features that establish the diagnosis. It was discovered globally in 1980, and it has two different types, namely, lymphocytic colitis (LC) and collagenous colitis (CC) [2,3]. Each of the two subtypes has distinct histologic characteristics, such as intraepithelial lymphocytosis, a significant inflammatory infiltration in the lamina propria, and subepithelial collagen band [4].

The clinical feature of MC is nonspecific and includes mainly a history of chronic watery, nonbloody diarrhea along with other gastrointestinal symptoms [1,4-6]. The disease course and severity range from mild intermittent symptoms to more progressive and chronic symptoms with dehydration and electrolyte derangement requiring hospitalization and proper management. Furthermore, while some individuals achieve spontaneous remission, others may require long-term maintenance treatment [7]. Increased awareness and knowledge of this disease in the past few decades has resulted in early diagnosis and proper management [8]. Despite being deemed a benign condition with no increase in mortality, as is the scenario with other inflammatory bowel diseases (IBDs), MC can lead to a significant negative impact on an individual’s quality of life.

## Review

Epidemiology

In the late 20th century, multiple studies from the America and European continents revealed a substantial increase in the prevalence of MC [9], whereas more recent statistics indicate that the incidence rates have plateaued [10,11]. Based on previous studies, the incidence of the collagenous type is estimated to be between 2 and 10.8 per 100,000 per year, and the incidence of the lymphocytic type is between 2.3 and 16 per 100,000 per year, with a greater incidence in northern Europe and North America [7,12-14]. The average age upon diagnosis is 65 years old [1]. Moreover, the diagnosis of MC occurs in around one-quarter of individuals before the age of 45. On the other hand, it has seldom been noticed in children [15-17]. MC has a female gender predominance [7,13,18]. Female predominance seems to be more evident in collagenous colitis than in lymphocytic colitis (female-to-male incidence rate ratios, 3.0 and 1.9, respectively) [10]. At present, Denmark has the greatest incidence, with around 16 collagenous cases per 100,000 people-years and around 11 lymphocytic cases per 100,000 people-years [19].

Risk factors

Several risk factors have been determined to have a significant impact on the development of MC, some of which are modifiable and others are not. The likelihood of getting the disease is higher in females, particularly in collagenous colitis as opposed to lymphocytic colitis, when comparing the two types [2].

Another major risk factor for MC includes active smoking. There exists evidence indicating a confirmed association between smoking and the pathogenesis of MC and adverse clinical outcomes [7,20,21]. When Yen et al. looked at 340 people with MC, they found that smoking cigarettes, whether in the past or at present, made the likelihood of getting the disease much higher [21]. In addition, smokers might develop the disease more than 10 years earlier than non-smokers [22].

Moreover, the presence of any personal medical background of autoimmune disorders, such as rheumatoid arthritis, diabetes mellitus, thyroid disorders, or celiac disease, is regarded as a significant risk factor [7,23-25]. Furthermore, the use of certain medications is considered one of the most common etiologies for MC [26,27]. For instance, the utilization of nonsteroidal anti-inflammatory drugs (NSAIDs), such as aspirin; proton pump inhibitors (PPIs), such as lansoprazole; and selective serotonin reuptake inhibitors are the major precipitant medications to cause the disease. In addition, various medications with different mechanisms of action including beta-blockers, angiotensin-converting enzyme inhibitors, and angiotensin receptor blockers or statins have been reported in previous studies [10,26,27]. Duloxetine, an antidepressant that inhibits the reuptake of serotonin and norepinephrine, has been associated with MC [28].

Pathophysiology

The etiology of MC remains uncertain; however, it is probably a complex process influenced by various factors, including mucosal immunological reactions in response to luminal factors in people with a hereditary predisposition [29]. The association between the development of MC and genetic predisposition remains uncertain. Nevertheless, there have been documented instances within families [4,7,30]. It is noteworthy that individuals within the same family exhibited the development of either lymphocytic or collagenous colitis, providing evidence for a shared underlying pathophysiological mechanism. Previous studies have demonstrated a correlation between MC and HLA-DQ2 or DQ1,3. In addition, HLA-DR3DQ2 haplotype and tumor necrosis factor 2 allele carriers were observed more frequently in MC patients compared to normal populations [31,32].

Meanwhile, the dense collagen band in collagenous colitis may be caused by a defective collagen metabolism. The dominant subepithelial matrix deposition has a major role in enhancing the expression of the principal fibrogenic genes, metalloproteinase inhibitor, and procollagen I by myofibroblastic cells along with poor fibrinolysis [30,33]. Moreover, an increased expression of transforming growth factor (TGF) beta-1 has been linked with collagen storage in tissues of individuals diagnosed with collagenous colitis [34].

One potential hypothesis is that an impairment in the function of the epithelial barrier and the presence of luminal substances could result in an elevated permeability of antigens and bacteria across the mucosal layer, thus leading to immune dysregulation and the manifestation of intestinal inflammation observed in cases of MC [35]. The proposed hypothesis that supported the involvement of certain bacteria in the pathogenesis of MC was obtained from the improvement of symptoms and histology following a treatment with a bismuth course in patients diagnosed with lymphocytic colitis and after a successful treatment of *Helicobacter pylori* in patients diagnosed with collagenous colitis [1,24,36]. The species that were observed in the literature along with *H. pylori* were *Campylobacter* species, *Clostridium difficile*, and *Yesinia* species [24]. Nevertheless, this theory lacks reliability since the administration of budesonide, a local immunosuppressive therapy, has proven to be quite effective in treating MC [8,24,37].

Autoimmunity has been identified as a potential factor in the pathogenesis and development of MC. This correlation had been identified on several studies. One of the largest studies looked at this correlation was a case control study conducted in Denmark by Wildt et al. in 2021 [38]. It involved 155,910 controls and 15,597 patients diagnosed with MC and documented a notable association between autoimmune disease and MC, particularly celiac disease, Crohn's disease, and ulcerative colitis. The study also revealed a higher occurrence of autoimmune illness in individuals with collagenous colitis as opposed to those with lymphocytic colitis. An additional study of 103 patients diagnosed with MC revealed that 39% (n=40) of the patients had a coexisting autoimmune disease. Particularly, there was no significant variation observed in the prevalence of autoimmune diseases between patients diagnosed with collagenous colitis and those diagnosed with lymphocytic colitis. Among the population of interest, *Hashimoto thyroiditis *emerged as the most observed autoimmune illness, affecting 14 individuals, accounting for 35% of the cases. This was followed by rheumatoid arthritis, which was present in seven patients, constituting 17.5% of the sample. Similarly, Sjogren's syndrome was also identified in seven patients, representing another 17.5% of the total cases [39].

The understanding of the relationship between bile acid malabsorption and MC is currently limited, mostly due to the complexity of the physiology and metabolism of bile acids inside the intestinal tract [40-42]. Furthermore, the intestinal tract has several receptors that have the ability to bind bile acids. These receptors play a significant role in physiological processes and contribute to the complexity of analyzing bile acid physiology in the colon. The manifestation of diarrhea linked to bile acid malabsorption can be attributed to either an augmentation in the production of bile acids or a reduction in their absorption in the terminal ileum. Furthermore, the conversion of primary bile acids to secondary bile acids is based upon the presence of specific bacteria within the colon. Any modifications in the intestinal bacteria could potentially impact the composition of bile acids within the colon. Bile acids can potentially induce MC in certain persons. Conversely, these acids may potentially exert a secondary influence by exacerbating diarrhea symptoms in individuals diagnosed with pre-existing MC [43]. The etiology of bile acid malabsorption in individuals diagnosed with MC is believed to involve multiple pathways. Certain patients with MC have evidence of villous atrophy and inflammation in the ileum, which may result in bile acid malabsorption and heightened levels of bile acids in the colon [44,45]. The presence of the ileum in these individuals may indicate the potential dissemination of the disease from the colon to the ileum. Moreover, it is plausible that a subset of these individuals may possess undetected celiac disease, a condition that affects the ileum [25]. These individuals may experience reduced absorption of bile in the terminal ileum, leading to elevated levels of bile acids in the colon. This, in turn, could potentially impact the onset and duration of diarrhea [46].

Clinical presentation

The typical presentation of the disease is chronic watery, nonbloody diarrhea. It is often present during the night and early morning and is frequently associated with fecal urgency, incontinence, and abdominal pain [1,47]. In the majority of cases, the symptoms develop in a gradual and progressive course, but around 40% of patients might develop sudden onset symptoms [47]. The mean frequency of diarrhea episodes typically ranges from three to eight loose stools per day, and in rare severe situations, it could reach up to 15 episodes per day [1,48]. Furthermore, the diarrhea might be accompanied by abnormal weight loss or other extraintestinal symptoms, such as uveitis, arthralgia, or arthritis. Collagenous colitis appears to be a more severe form of bowel inflammation than lymphocytic colitis, which tends to manifest earlier in life [49]. There exists a third entity characterized by patients exhibiting typical clinical symptoms but lacking the precise histological markers associated with either the collagenous or lymphocytic subtype. This entity is called incomplete MC or MC not otherwise specified (NOS) [50,51]. It is crucial for primary healthcare providers to offer an early referral to gastroenterologist if they have any patient with unexplained chronic diarrhea for proper evaluation and diagnosis, which will have a great positive impact on the patient's quality of life and disease prognosis.

Diagnosis

The presence of chronic diarrhea and related associated symptoms should raise suspicions for the potential diagnosis of MC, especially in middle-aged and older persons. The establishment of a diagnosis is challenging and requires ruling out another differential diagnosis of chronic diarrhea. The laboratory workup should include a routine stool culture or polymerase chain reaction (PCR) testing for different possible causative bacterial organisms, such as *Campylobacter*, *Shigella*, *Salmonella*, *Yersinia*, *Clostridioides difficile*, and *Escherichia coli* O157:H7 [39]. In addition, parasitic infections need to be ruled out by performing stool for ova and parasites along with the Giardia stool antigen [39]. Celiac serologies are also recommended to exclude the possibility of celiac disease [4,25].

The primary diagnosis method for this condition involves obtaining histologic samples from various sites (specifically, two to four biopsies of colon segments) during a colonoscopy procedure. This approach is essential since mucosal inflammation may not always be apparent during colonoscopy. However, it is important to highlight that there are certain nonspecific macroscopic signs of colitis, which might be indicative of the presence of underlying MC [5]. These signs include a slightly swollen mucosa, friability, exudative lesions, and hyperemic bowel wall [5,52].

**Figure 1:**
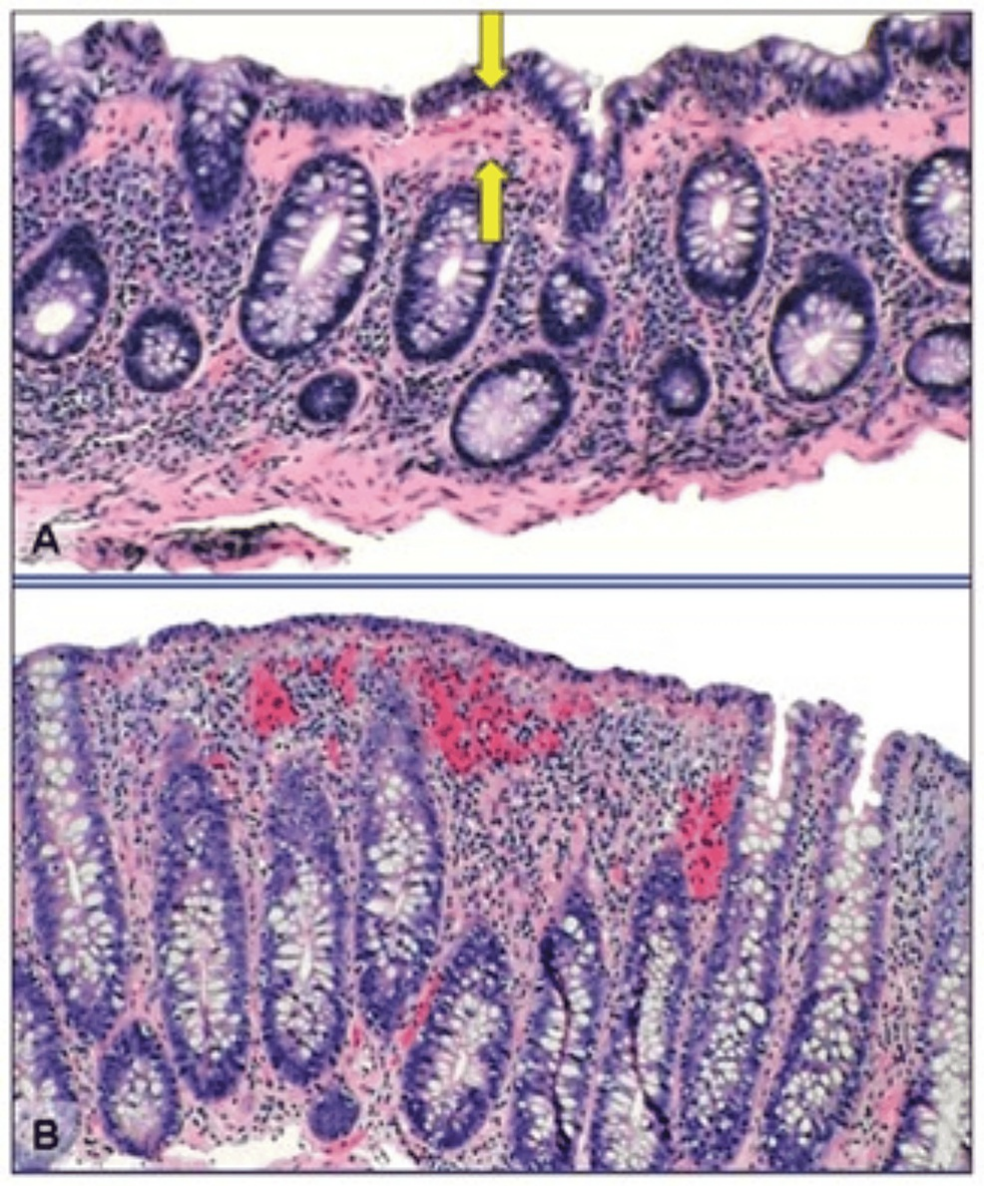
Histological features of microscopic colitis. (A) Collagenous colitis is associated with an increased lymphocytic infiltrate of the lamina propria and intraepithelial lymphocytosis. There is a markedly thickened (>10 μm) collagen band underlying the colonic epithelial cells (arrows). (B) Lymphocytic colitis is associated with an increased lymphocytic infiltrate of the lamina propria and intraepithelial lymphocytosis. The collagen band underlying the colonic epithelial cells is normal in width (<10 μm) [58].

Colonoscopy with subsequent biopsies from the right- and left-sided colon is generally considered to be a safe procedure for patients who have been diagnosed with MC. The incidence of perforations has been documented in individuals with substantial collagen accumulations, sometimes referred to as fractured colon. However, such occurrences are infrequent [53]. The justification for conducting a colonoscopy instead of a narrower assessment of the left colon using flexible sigmoidoscopy lies in the fact that MC has the potential to exhibit patchy characteristics. Furthermore, there is a gradual decrease in the degree of histologic alterations as one moves from the proximal to the distal colon [6,19]. The inflammatory cellular activity in collagenous and lymphocytic colitis exhibits similarities, characterized predominantly by mononuclear infiltrates in the lamina propria with eosinophils. Nevertheless, specific histologic characteristics are taken into consideration to differentiate between collagenous and lymphocytic colitis [3,17,18,48]. The colonic mucosa in the collagenous type showed evidence of a subepithelial collagen band of at least 10 micrometers in diameter between crypts [54]. Meanwhile, lymphocytic colitis is characterized by at least 20 intraepithelial lymphocytes per 100 surface epithelial cells with no evidence of crypt architecture distortion [55-57]. The histological feature of MC is shown in Figure 1 [58].

Management

The main objective of managing individuals diagnosed with MC is to accomplish clinical remission (characterized by a reduction in daily stool frequency to one to two bowel motions and the absence of watery stool over the course of seven days) to enhance the overall quality of life experienced by the patients. The currently available management options include a range of interventions, starting with avoidance of triggering factors followed by a variety of pharmaceutical treatments targeting conservative treatment of diarrheal episodes. In cases where initial conservative measures fail to control symptoms, more potent lines of management, such as oral corticosteroids, immunomodulators, or biologic agents, can be introduced. Finally, in refractory and severe cases, surgical interventions might have a role as the last management option.

Lifestyle Modifications

The initial management strategy involves the identification of modifiable factors, such as smoking and drug-induced cases. Patients must be counseled regarding the significant role of smoking in disease pathogenesis and progression [21]. It is crucial for healthcare providers to actively promote tobacco cessation among patients and advocate for their participation in national smoking cessation programs.

In addition to smoking cessation, it is necessary to review the patient’s medication list as an essential part of the initial management given its potential for reversibility and avoid possible offending medications, such as nonsteroidal anti-inflammatory drugs, proton pump inhibitors, antidepressant medications, beta-blockers, angiotensin-converting enzyme inhibitors, angiotensin receptor blockers, or statins. These medications need to be screened, and it is recommended to discontinue or provide a safer alternative if possible [59].

Antidiarrheal Medications

The primary objective of antidiarrheal medications is to reduce the frequency of nighttime attacks. Loperamide is considered the preferred therapeutic agent [37,39,60]. Loperamide is a synthetic phenylpiperidine opioid with a high degree of lipophilicity. Loperamide functions as an agonist of the mu-opioid receptor. At recommended dosages, loperamide exerts its effects by interacting directly with the mu-opioid receptors located in the muscles of the colon. This interaction leads to a reduction in the duration of time for substances to pass through the colon, an inhibition of peristalsis, a reduction in electrolyte loss, and an optimization of rectal activities [39,60,61].

Bismuth

Bismuth subsalicylate and bismuth subcitrate enemas showed some benefits in treating MC in a few limited studies. In a prospective study including 12 patients with MC who were treated with bismuth subsalicylate 262 mg daily for eight weeks, a significant clinical remission was observed in 11 patients, with a histological response in nine patients [62]. The average time to response was two weeks, and almost three quarters of the patient population remained in remission at 28 months' follow-up [62].

Budesonide

For patients who exhibit active diseases, characterized by a minimum of three stools daily or at least one watery stool daily, or those who experience persistent diarrhea despite the administration of antidiarrheals, it is advisable to incorporate oral budesonide at a dosage of 9 mg per day for a duration of six to eight weeks [4,60,63]. Budesonide is a potent and effective glucocorticoid that has a broad spectrum of anti-inflammatory properties. The administration of budesonide orally typically results in primarily local effects, with minimal systemic exposure as a result of its pharmacokinetic action and extensive first-pass effect [39,47,60,61]. According to the current guidelines, it is recommended that budesonide be utilized initially for treatment induction and to maintain remission in MC [4,8,39,47,60,61]. The treatment's success rate for budesonide was 88% in collagenous colitis, whereas only one-third of patients treated with a placebo had a significant clinical response based on Kafil et al.'s study [64]. Meanwhile, the remission rate for the budesonide-treated group in lymphocytic colitis was 84% compared with less than half of the total patients who were treated with a placebo [65]. There was also a strong evidence of histological response based on Chande et al.'s study [65].

Cholestyramine

Cholestyramine is a pharmaceutical agent classified as a bile-acid binder, which has demonstrated efficacy in the management of diarrhea in individuals presenting with bile acid malabsorption [66]. It can be used in patients with mild, persistent diarrhea despite budesonide, as an adjunctive medication with loperamide, and the recommended dosage of cholestyramine is 4 grams every six hours.

Aminosalicylates

Aminosalicylates, such as mesalamine, appear ineffective in treating collagenous colitis and lymphocytic colitis [1,67]. In an eight-week randomized clinical trial, a total of 92 patients diagnosed with active collagenous colitis were administered either oral budesonide (9 mg daily), mesalamine (3 grams daily), or a placebo. The study findings revealed that the remission rates observed with mesalamine were similar to those shown with the placebo [68]. Maintenance therapy has not been investigated. In the same study, 57 patients with active lymphocytic colitis were randomized to receive therapy with budesonide, mesalamine (at a dosage of 3 grams per day), or a placebo for a duration of eight weeks. The results indicated that there was no statistically significant difference in the rates of clinical and histologic remission between the mesalamine group and the placebo group [68].

Probiotics

Taking into consideration the potential role of gut microflora in the pathogenesis of MC, one study evaluated the effectiveness of probiotics as a possible treatment option. Wildt et al. studied the association of *Lactobacillus acidophilus* LA-5 and *Bifidobacterium* species in treating MC and found no superiority in comparison to a placebo [69]. Further studies are needed to assess the effectiveness of probiotics in patients with MC.

Immunomodulators and Biological Agents

Immunomodulators and biological agents are administered to selected individuals who are glucocorticoid-resistant or intolerant. Limited evidence from small case series and retrospective studies suggests that immunomodulators and biological agents can induce remission in refractory cases of MC. Azathioprine and 6-mercaptopurine are immunomodulators of choice, and they can achieve clinical remission up to 28% and 46%, respectively, based on Munch et al.'s study [70]. Biological therapies, such as anti-tumor necrosis factor drugs (infliximab and adalimumab) and integrin receptor antagonists (vedolizumab), are the agents to be considered in such clinical scenarios [71-74].

Surgical interventions

When medical treatments fail to induce remission, surgery may be considered for refractory and severe cases. The preferred procedure is diverting ileostomy, but a total colectomy may also be considered [60]. The evidence for surgical intervention in refractory came from few case reports and case series. For example, a middle-aged man who was diagnosed with collagenous colitis for five years and failed multiple lines of medical therapy eventually underwent total protocolectomy followed by ileal pouch anal anastomosis, and on the two-year follow-up after the surgery, he had a reasonable control of disease symptoms [75]. Once again, it is observed that most patients tend to exhibit positive responses to the medical management of MC. Consequently, surgical interventions should only be offered for individuals who have failed all alternative therapies, eliminated all other potential precipitating factors, and are experiencing severe and incapacitating diarrhea.

Disease monitoring and long-term assessment

Disease monitoring and long-term assessment are considered essential components to ensure good compliance to the management plan and to assess the need for an alternative plan in the case of disease relapse. Regular follow-up is usually given to the patient upon starting the treatment until clinical remission is achieved. After that, the aim of the primary physician will be to maintain the remission over time with an annual assessment and easy clinic booking in the case of flaring-up of symptoms. Moreover, a close monitoring of any possible treatment side effect is crucial. For example, the patient must be provided with calcium and vitamin D supplements in the case of long-term corticosteroid therapy.

Disease prognosis

MC might improve within week or few months with interventions. In some cases, spontaneous recovery has been noticed. Clinical relapse after a successful treatment were observed to range from 30% to 60% based on few studies; therefore, the long-term follow-up is very crucial. In the literature review, there were a few studies that looked at the natural history and disease prognosis after proper management. Fernández et al. examined 37 patients with collagenous colitis and 44 patients with lymphocytic colitis in a three-year prospective study, which involved the administration of a different line of management, and found that two-third of the targeted group maintained long-term remission while the remaining third had a relapse on the long-term follow-up [76]. There is currently no evidence suggesting a greater risk of developing colorectal cancer in individuals with MC. Furthermore, the disease transition from collagenous colitis to lymphocytic colitis is infrequent.

Future prospectives for research and awareness on MC

Although the prevalence of MC as a common cause of chronic diarrhea, particularly among the older population, is growing, there is a need to enhance awareness among healthcare professionals in countries where the reported incidence is relatively low. The understanding of the function of the gut microbiota or specific gene profiles has the potential to create opportunities for the development of novel evidence-based therapeutic approaches aimed at minimizing disease progression and flares and potentially achieving a curative outcome. The current therapeutic interventions for MC exhibit some constraints, hence necessitating the exploration of novel therapy modalities. To date, the primary objective in the care of MC has been to address symptoms and enhance the quality of life for patients in the immediate term. There is potential for additional medications, beyond those commonly used in the treatment of traditional types of IBD, to have a positive impact on reducing relapse risk and sustaining remission over an extended period of time. Furthermore, there remains uncertainties regarding the appropriate categorization of collagenous colitis and lymphocytic colitis as distinct entities. An area that requires attention is the identification and verification of noninvasive biomarkers, such as fecal calprotectin or mucosal and fecal neutrophil gelatinase-associated lipocalin, with the aim of serving as potential indicators for MC. These biomarkers have the potential to evaluate and anticipate the clinical activity of the disease.

## Conclusions

MC is a gastrointestinal condition distinguished by gastroenterologists in their practice and characterized mainly by chronic persistent watery diarrhea. The prevalence and subsequent recognition of this disease have been on the rise. Maintaining a heightened level of suspicion is of utmost importance when evaluating individuals from specific demographics who exhibit symptoms of persistent diarrhea. This is particularly relevant for middle-aged women and the elderly. In such cases, it is imperative to make appropriate referrals for lower gastrointestinal endoscopy with biopsy when deemed necessary. There are two histological distinctive types, collagenous colitis and lymphocytic colitis. Although lifestyle modifications, such as smoking cessation, and a comprehensive medication history review to eliminate potential drug-induced etiology, have proven advantageous, budesonide has been proven by various international societies as the primary treatment for MC. Although budesonide has demonstrated high rates of success, there exists a potential for recurrence. The administration of low-dose maintenance budesonide is an effective approach in treating cases of relapsing MC cases; however, additional data are required to further support these findings. The literature discusses several alternative pharmaceutical options; although specific options have minimal supporting evidence, others lack any empirical data.
